# Progression risk stratification with six-minute walk gait speed trajectory in multiple sclerosis

**DOI:** 10.3389/fneur.2023.1259413

**Published:** 2023-10-04

**Authors:** Myla D. Goldman, Shanshan Chen, Robert Motl, Rylan Pearsall, Unsong Oh, J. Nicholas Brenton

**Affiliations:** ^1^Department of Neurology, Virginia Commonwealth University, Richmond, VA, United States; ^2^Department of Biostatistics, Virginia Commonwealth University, Richmond, VA, United States; ^3^Department of Kinesiology & Nutrition, University of Illinois at Chicago, Chicago, IL, United States; ^4^College of Arts and Sciences, University of Virginia, Charlottesville, VA, United States; ^5^Department of Neurology, Division of Pediatric Neurology, University of Virginia, Charlottesville, VA, United States

**Keywords:** multiple sclerosis, six-minute walk, progression, growth mixture model, gait speed trajectory

## Abstract

**Background:**

Multiple Sclerosis (MS) disease progression has notable heterogeneity among patients and over time. There is no available single method to predict the risk of progression, which represents a significant and unmet need in MS.

**Methods:**

MS and healthy control (HC) participants were recruited for a 2-year observational study. A latent-variable growth mixture model (GMM) was applied to cluster baseline 6-min walk gait speed trajectories (6MW^GST^). MS patients within different 6 MW^GST^ clusters were identified and stratified. The group membership of these MS patients was compared against 2-year confirmed-disease progression (CDP). Clinical and patient-reported outcome (PRO) measures were compared between HC and MS subgroups over 2 years.

**Results:**

62 MS and 41 HC participants completed the 2-year study. Within the MS cohort, 90% were relapsing MS. Two distinct patterns of baseline 6 MW^GST^ emerged, with one cluster displaying a faster gait speed and a typical “U” shape, and the other showing a slower gait speed and a “flattened” 6 MW^GST^ curve. We stratified MS participants in each cluster as low- and high-risk progressors (LRP and HRP, respectively). When compared against 2-year CDP, our 6 MW^GST^ approach had 71% accuracy and 60% positive predictive value. Compared to the LRP group, those MS participants stratified as HRP (15 out of 62 MS participants), were on average 3.8 years older, had longer MS disease duration and poorer baseline performance on clinical outcomes and PROs scores. Over the subsequent 2 years, only the HRP subgroup showed a significant worsened performance on 6 MW, clinical measures and PROs from baseline.

**Conclusion:**

Baseline 6 MW^GST^ was useful for stratifying MS participants with high or low risks for progression over the subsequent 2 years. Findings represent the first reported single measure to predict MS disease progression with important potential applications in both clinical trials and care in MS.

## Introduction

Multiple Sclerosis (MS) is a neuroinflammatory and degenerative disorder characterized by both relapses and progression. Patients experience notable variation in the degree of progression independent of relapse activity over their disease course. Progression rates may vary among individuals, clinical phenotypes, and by the approach to measuring progression (e.g., single outcome vs. composite measures). Predicting whether a patient is likely to progress over the short or long term is challenging. Several large cohort studies have attempted to develop prediction models for MS prognosis, however, none of the clinical variables are predictive of progression in primary progressive MS [e.g., age onset, gender, type of first symptoms, and early Expanded Disability Status Scale (EDSS)] (1) and relapse-remitting MS (e.g., age onset, gender except onset of secondary progression) (2). The inability to identify an individual’s risk for progression may contribute to the disappointing results of clinical trials in progressive MS (3, 4). On-study progression rates have been notably low, even in the placebo arms and despite efforts to recruit patients who had demonstrated “progression” pre-trial based on traditional clinical outcome measures. Although traditionally conceptualized as a delayed aspect of disease in relapsing MS patients, we now recognize that disease progression begins early in the disease course, even in those with a relapsing phenotype. This concept of progression in relapsing patients, coined as “progression independent of relapse activity (PIRA)”, has been reported in several studies focused on relapsing MS patients (5). However, in relapsing MS patients, the risk of progression over a 2-year study is small (4–24%), which further limits our understanding of the impact of MS treatments on progression in those with a relapsing course. Predicting the risk of MS progression would have significant value both clinically and in future therapeutic trials.

While a complete understanding of factors driving progression in MS is lacking, one posited driver of progression is the ultimate demise of demyelinated axons. Denuded or insufficiently-remyelinated axons are vulnerable to oxidative stress and mitochondrial dysfunction, leading to delayed and eventual degeneration (6–8). Physiologically, a denuded axon would have conduction delay and/or failure with prolonged activation, as would occur during a prolonged walking test, such as the 6-min walk (6 MW). We have previously shown that by capturing the deceleration pattern, parameters of the 6 MW gait speed trajectory (6MW^GST^) are more sensitive at differentiating MS patients from healthy controls (9). In this paper, we evaluated if a baseline 6 MW^GST^ could be utilized to stratify MS patients into groups with high and low risks for progression measured by clinical and patient-reported outcomes at a 2-year timepoint.

## Methods

### Participants

MS participants and healthy controls (HC) were recruited for a prospective observational 2-year longitudinal study between 2010 and 2015. This study was approved by the University Institutional Review Board for Health Sciences Research. All participants signed informed consent before study-related procedures and were seen every 6 months for 2 years. Each visit included 6 MW, clinical, and PRO measures. MS participants were identified through the Neurology outpatient clinic and had a diagnosis of confirmed MS (10) with either a relapsing or progressive subtype. Inclusion criteria included age 18–64 years and the ability to ambulate for 6 minutes. Exclusion criteria included: MS relpase or steroid use within 90 days, neurological impairment from other diagnoses, orthopedic limitations, morbid obesity (BMI > 40), and/or known cardiac or respiratory disease. Medications with the potential to impact fatigue or outcome measures (e.g., dalfampridine or modafinil) were held 48 hours before visits.

### Clinical assessment and disability measures

Baseline demographics, smoking exposure (by pack-years), medical history, and medications were documented. MS-related disability was assessed using the Expanded Disability Status Scale (EDSS) (11) by a certified Neuro-status examiner [MDG]. The Multiple Sclerosis Functional Composite (MSFC) (12) included timed 25-foot walk (T25FW), 9-hole peg test (9HPT), paced auditory serial addition test (PASAT), and symbol digit modalities test (SDMT) (13).

### Six-minute walk (6 MW) test

We administered 6MW tests in a 175-foot hallway using the validated script by Goldman et al. (14), instructing subjects to *walk as far and as fast as possible.* Visits occurred at 9:00 a.m. to eliminate any possible time-of-day variability on 6 MW testing. Minute-by-minute 6 MW distance was measured using a surveyor measuring wheel (Stanley MW50, New Briton, CT). Minutes during 6 MW were indexed as 0, 1, 2, 3, 4, and 5. Visits over 2 years were indexed as 0, 1, 2, 3, and 4.

### Physical activity counts

We measured physical activity using ActiGraph accelerometers (GT2X+; ActiGraph, FL, United States) which were worn on non-dominant hips for 7 days while awake, except during swimming or bathing. Wear time compliance was assessed, and analysis included those with ≥10 h/day for at least 3 valid days.

### Patient reported outcomes (PROs)

*Short Form 36 (SF-36)* assessed health-related QoL (15), with higher scores indicating better QoL. *MS Impact Scale (MSIS-29)* measured MS-related disability (16), with 20 questions on physical function and 9 on psychological function. *Modified Fatigue Impact Scale (MFIS)*, a 21-item instrument, assessed the impact of fatigue on functioning (17), where a higher score indicates greater fatigue impact. *Fatigue Severity Survey (FSS)*, a 9-item validated survey measuring fatigue with a higher score indicating greater fatigue severity (18).

### Confirmed disease progression (CDP)

CDP was defined as having any one of the following criteria in any two out of four follow-up visits, or at any single visit at 18 or 24 months: 1) increased EDSS ⩾1.0-point increase from a baseline score of ⩽5.5 or a ⩾0.5-point increase from a baseline score of ⩾6.0, and/or a ⩾20% increase in T25FW or 9HPT score (19).

## Statistical power & analysis

*A priori* sample size of 64 was calculated to detect a difference of 0.75 (Cohen’s D) in the total distance of baseline 6 MW within the MS sample, with 80% power and a two-sided significance level of 0.05. Subsequent to data collection, we elected to use more efficient tests (mixed-effects models) for 6 MW data analysis beyond the total distance of 6 MW, and were able to gain more power in our statistical tests.

**Figure 1:**
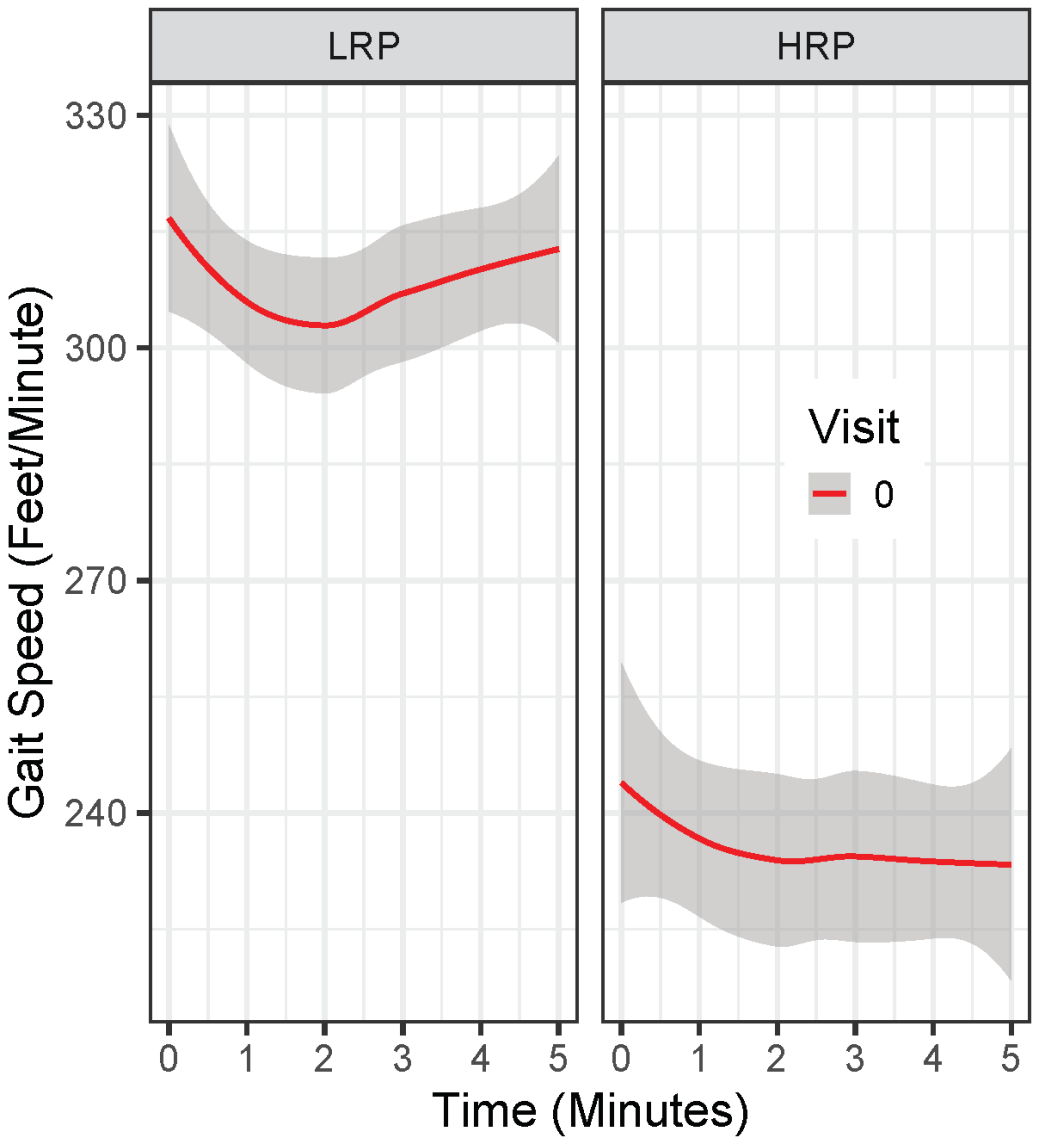
Baseline 6 MW gait speed trajectory in Low Risk Progressor (LRP) and High Risk Progressor (HRP) group. Visit 0 = baseline visit.

Analysis was done in R Studio (R version 4.1.1, RStudio Inc., Boston, Massachusetts). First, we identified potential MS subgroups by fitting growth mixture models (GMM) to the minute-by-minute 6 MW data at the baseline visit. GMM is a technique for identifying unobserved subppopulations by clustering similar longitudinal trajectories into groups and examines differences in the clustered trajectories. Upon visualizing the temporal trends of 6 MW data, we chose quadratic curves to capture the temporal effect in the GMM models. Since BMI can be associated with 6 MW performance in MS (20–22) and non-MS populations (23, 24), we adjusted GMM models for BMI. We fit five GMM models with varying numbers of latent classes (i.e., 1–5) and selected the GMM model with the best model fit (two latent classes) – evaluated by Akaika information criteria (AIC) and Bayesian Information Criteria (BIC). All GMM models were fitted using the “hlme” function *via* maximum likelihood estimation in the R package “lcmm” (25). Model validity was checked by leave-one-subject-out cross-validation (CV) and 100 simulations. Among the CV folds and simulations, parameter estimates and class memberships were consistent, and the accuracy of classification was above 90% among the simulations, with all but three GMM models not converging given a maximum iteration of 6,000. Second, we applied the selected GMM model to baseline 6 MW^GST^ and identified MS participants within each group by estimating the posterior probability. We visualized the 6 MW^GST^ of each group (Figure 1). On average, one group of patients walked slower and failed to speed up by the end of 6 MW. We stratified this group as “High Risk Progressors (HRP),” and those who were not “High Risk Progressors” were stratified as “Low Risk Progressors (LRP).” Third, we further examined whether the two MS groups identified by GMM are merely due to gradation in 6 MW by comparing the two groups in demographics, clinical assessments, and PROs. Specifically, we fit linear mixed-effects (LME) models (complete-case analysis) to the longitudinal clinical outcomes from 5 visits (including 6 MW, EDSS, T25FW, 9HPT, PASAT, and SDMT), as well as the longitudinal PRO outcomes (including SF-36, MSIS-29, MFIS, and FSS), with a categorical variable indicating the two MS subgroups and HC. For the longitudinal 6 MW outcome, we adjusted for age, sex, smoking exposure, and BMI in the model, as well as the the linear and quadtric form of time and the linear form of visits. Both time and visits were modeled as continuous variables. Multiple comparisons with the false discovery rate controlled by the Benjamini-Honchberg procedure (26) were conducted to detect significant progression from baseline scores across clinical outcomes and PRO outcomes. Lastly, we compared the GMM + 6 MW^GST^method with other baseline variables (e.g., age, BMI, MSFC tests, total distance of 6 MW etc.) clustered by a two-cluster K-means algorithm. We evaluated area under the ROC curve (AUROC), accuracy, positive predictive values (PPV), negative predictive values (NPV), sensitivity, and specificity by comparing the identified HRPs of each clustering method against the CDP-defined progressors.

## Results

A total of 62 MS and 41 HC participants were enrolled. Baseline characteristics are presented in Table 1. Compared to HCs, MS participants were older and predominantly female, consistent with MS prevalence (27). MS participants had reduced performance on 6 MW and MSFC components, as well as reduced physical activity compared to HCs. At baseline, MS participants had a mild-to-moderate disability (EDSS 1.0–4.0). Over the 2-years we had good participant retention; total missed visits and/or loss to follow-up (withdrawl) were: 6, 12, 11 and 18% for the four follow-up visits.

**Table 1:** Baseline characteristics of all participants.

	Control (*N* = 41)	MS (*N* = 62)	Overall (*N* = 103)
**Age** (years)			
Mean (SD)	34.7 (12.0)	41.6 (8.92)	38.8 (10.8)
Median [Min, Max]	35.0 [18.0, 55.0]	42.0 [19.0, 55.0]	40.0 [18.0, 55.0]
**Sex**			
Male	12 (29.3%)	14 (22.6%)	26 (25.2%)
Female	29 (70.7%)	48 (77.4%)	77 (74.8%)
**Height** (cm)			
Mean (SD)	170 (8.13)	169 (9.90)	170 (9.29)
Median [Min, Max]	169 [154, 195]	169 [150, 188]	169 [150, 195]
**Weight** (kg)			
Mean (SD)	78.0 (33.5)	81.1 (25.5)	79.8 (28.7)
Median [Min, Max]	71.5 [46.7, 264]	77.0 [45.5, 230]	75.0 [45.5, 264]
**BMI**			
Mean (SD)	25.6 (5.49)	27.5 (5.18)	26.8 (5.36)
Median [Min, Max]	24.8 [16.6, 40.5]	26.6 [17.6, 38.6]	26.2 [16.6, 40.5]
**Gait speed 1^st^ minute** (feet/min)			
Mean (SD)	357 (44.5)	303 (56.6)	325 (58.2)
Median [Min, Max]	366 [247, 436]	306 [180, 417]	330 [180, 436]
**Gait speed 6^th^ minute** (feet/min)			
Mean (SD)	351 (49.0)	290 (53.9)	315 (59.8)
Median [Min, Max]	362 [232, 426]	294 [159, 391]	322 [159, 426]
**6MW Total distance** (feet)			
Mean (SD)	2080 (265)	1750 (320)	1880 (340)
Median [Min, Max]	2,140 [1,370, 2,530]	1760 [1,010, 2,320]	1930 [1,010, 2,530]
**Physical activity** (Counts)			
Mean (SD)	298k (153k)	197k (107k)	237k [136k]
Median [Min, Max]	193k [17.8k, 686k]	170k [53.6k, 604k]	189k [17.8k, 686k]

By clustering baseline 6 MW^GST^ of the 62 MS patients, the GMM approach identified two MS progressor groups. Baseline 6 MW^GST^ of these two groups are illustrated in Figure 1. One MS subgroup demonstrated a typical “U” shape (28), marked by an acceleration in the final minutes of the 6 MW (Figure 1). The other MS subgroup had a slower gait speed and a distinct “flattened” 6 MW gait speed trajectory curve. Thus, the 6 MW^GST^ analysis demonstrated two distinct patterns in our MS cohort. We stratified the first group as the “low risk progressors” (LRP, *n* = 47) and the other as the “high risk progressors” (HRP, *n* = 15). At baseline, HRPs had a longer MS disease duration (17.5 ± 8.5 vs. 12.6 ± 6 years, *p* = 0.039), were older (44.5 ± 8 vs. 40 ± 9 years, *p* = 0.51), higher smoking exposure (8.3 ± 15.6 vs. 3.5 ± 6.8 pack-years), and had lower BMI (25.4 ± 5.0 vs. 28.2 ± 5.1, *p* = 0.57) compared to the LRPs (Table 2). In addition, at baseline, the HRPs had a higher EDSS score (3.1 ± 0.7 vs. 2.3 ± 0.8, *p* = 0.13), poorer performance on clinical outcome measures (6 MW, T25FW, 9-HPT, SDMT, and PASAT), and worse PRO scores (FSS, MSIS-29, MFIS) than the LRPs. Other baseline features of these two MS progressor groups are outlined in Table 2.

**Table 2:** Baseline characteristics of the GMM method identified MS progressor subgroups.

	Low risk progressors (LRP) (*N* = 47)	High risk progressors (HRP) (*N* = 15)
**Age** (years)		
Mean (SD)	40.7 (8.97)	44.5 (8.18)
Median [Min, Max]	41.0 [19.0, 55.0]	46.0 [32.0, 55.0]
**Sex**		
Male	11 (23.4%)	3 (20.0%)
Female	36 (76.6%)	12 (80.0%)
**Race**		
Other	1 (2.1%)	1 (6.7%)
Black	6 (12.8%)	2 (13.3)
White	40 (85.1%)	12 (80.0%)
**MS type**		
Relapse and remitting	43 (91.5%)	13 (86.7%)
Primary progressive	2 (4.3%)	2 (13.3%)
Secondary progressive	2 (4.3%)	0 (0%)
**MS treatment**		
IFNB-1a IM	34 (72.3%)	12 (80%)
IFNB-1b	0 (0%)	1 (6.7%)
Glatiramer acetate	8 (17.0%)	1 (6.7%)
IFNB-1a SQ	2 (4.3%)	1 (6.7%)
Natalizumab	1 (2.1%)	0 (0%)
Fingolimod	2 (4.3%)	0 (0%)
**Disease duration** (years)		
Mean (SD)	12.6 (5.83)	17.5 (8.42)
Median [Min, Max]	13.0 [3.00, 26.0]	16.0 [8.00, 35.0]
**Smoking exposure** (pack-years)		
Mean (SD)	3.45 (6.77)	8.27 (15.6)
Median [Min, Max]	0 [0, 24.0]	0 [0. 54.0]
**Height** (cm)		
Mean (SD)	169 (10.5)	169 (8.55)
Median [Min, Max]	167 [151, 188]	169 [150, 183]
**Weight** (kg)		
Mean (SD)	83.3 (26.8)	73.6 (18.9)
Median [Min, Max]	77.2 [51.5, 230]	70.0 [45.5, 112]
**BMI**		
Mean (SD)	28.2 (5.11)	25.4 (4.99)
Median [Min, Max]	27.8 [20.0, 38.6]	23.1 [17.6, 35.3]
**EDSS**		
Mean (SD)	2.30 (0.78)	3.13 (0.67)
Median [Min, Max]	2.00 [1.00, 4.50]	3.00 [2.00, 4.00]
**9-Hole Peg** (seconds)		
Mean (SD)	20.2 (4.73)	24.6 (5.45)
Median [Min, Max]	19.0 [14.5, 47.0]	22.9 [19.5, 39.5]
**T25FW** (seconds)		
Mean (SD)	4.04 (0.732)	5.41 (1.09)
Median [Min, Max]	3.94 [2.58, 6.00]	5.19 [4.04, 8.37]
**SDMT**		
Mean (SD)	56.9 (13.7)	44.3 (12.0)
Median [Min, Max]	58.0 [24.0, 81.0]	47.0 [15.0, 66.0]
**PASAT**		
Mean (SD)	51.9 (9.03)	45.8 (10.5)
Median [Min, Max]	55.0 [25.0, 60.0]	47.0 [25.0, 60.0]
**Gait speed in the 1^st^ minute** (feet/min)		
Mean (SD)	317 (44.9)	245 (34.7)
Median [Min, Max]	314 [218, 405]	245 [183, 302]
**Gait speed in the 6^th^ minute** (feet/min)		
Mean (SD)	313 (38.5)	234 (32.3)
Median [Min, Max]	311 [242, 382]	246 [175, 275]
**6MW Total distance** (feet)		
Mean (SD)	1860 (224)	1,420 (179)
Median [Min, Max]	1860 [1,450, 2,270]	1,460 [1,080, 1,650]
**MSIS-29 total**		
Mean (SD)	49.4 (19.4)	61.0 (26.1)
Median [Min, Max]	44.0 [30.0, 108]	56.0 [32.0, 132]
**SF-36 total**		
Mean (SD)	102 (6.10)	99.9 (7.29)
Median [Min, Max]	102 [89.0, 113]	100 [89.0, 112]
**MFIS total**		
Mean (SD)	28.9 (18.2)	42.0 (15.5)
Median [Min, Max]	27.0 [0, 66.0]	46.0 [14.0, 69.0]
**FSS total**		
Mean (SD)	20.5 (10.6)	30.1 (7.53)
Median [Min, Max]	19.0 [9.00, 42.0]	30.0 [14.0, 43.0]

**Figure 2:**
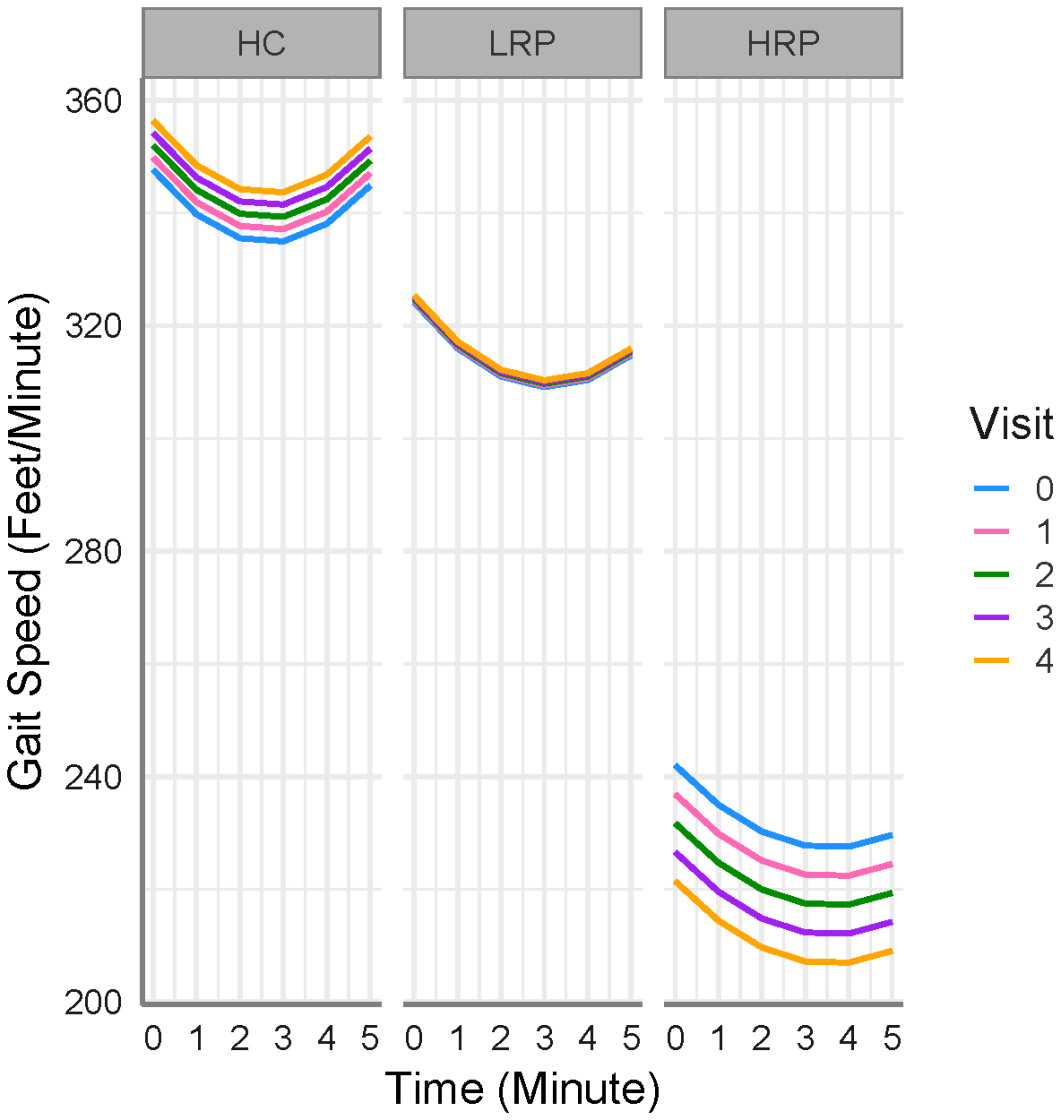
6 MW gait speed trajectories for all subjects. HC, healthy controls; LRP, low risk progressor; HRP, high risk progressor. Visit 0, baseline; Visit 1, 6 months; Visit 2, 12 months; Visit 3, 18 months; Visit 4, 24 months. Missing data were 7, 15, 16, 21% at four follow-up visits.

We compared longitudinal performance between HC and MS groups (HRP and LRP), using an LME model with the 6 MW^GST^ as the outcome (Table 3). After fitting the age, sex, BMI, and smoking exposure-adjusted model, only BMI was significantly associated with 6 MW. Thus, age, sex and smoking exposure were removed from the final model. We found that both MS groups walked significantly slower than HC with a baseline difference of 23 feet/min for LRP (*p* = 0.009) and 106 feet/min for HRP (*p* < 0.001). In addition, the HRPs decelerated at the 2nd to 5th minute more severely than HCs by 1, 2, 4, 5 feet/min^2^ and 0.1, 1, 2, 3 feet/min^2^ relative to LRPs. Moreover, when compared longitudinally, the HRPs had a significant worsening of 6 MW gait speed over time (5 feet/min reduction each 6-month visit, *p* < 0.001). In contrast, the LRPs demonstrated no significant decrease in 6 MW over time, and HCs increased 6 MW gait speed over subsequent visits (2 feet/min/visit, *p* = 0.005). The findings of the LME modeling are illustrated in Figure 2, which demonstrates the longitudinal 6 MW^GST^ of HC and two MS subgroups.

**Table 3:** Results from the LME model with 6 MW gait speed as the outcome.

Fixed effects	Outcome: 6 MW gait speed		
	Estimates	CI	*p*
(Intercept)	347.65	335.22–360.07	<0.001
LRP	-23.48	-40.98–-5.98	0.009
HRP	-105.63	-129.80–-81.47	<0.001
Time (0-5)	-9.74	-11.36–-8.12	<0.001
Time^2^	1.84	1.56–2.11	<0.001
Visit (0-4)	2.18	0.65–3.71	0.005
BMI	-3.65	-5.12–-2.17	<0.001
LRP * Time	0.01	-2.19–2.22	0.990
HRP *Time	1.56	-1.63–4.75	0.337
LRP* Time^2^	-0.27	-0.65–0.11	0.166
HRP *Time^2^	-0.70	-1.24–-0.15	0.013
LRP * Visit	-1.88	-3.95–0.20	0.076
HRP * Visit	-7.32	-10.38–-4.26	<0.001

LRP, low risk progressor; HRP, high risk progressor; BMI, body mass index; Time, Minute index during 6 MW; Visit 0, baseline; Visit 1, 6 months; Visit 2, 12 months; Visit 3, 18 months; Visit 4, 24 months.

**Figure 3:**
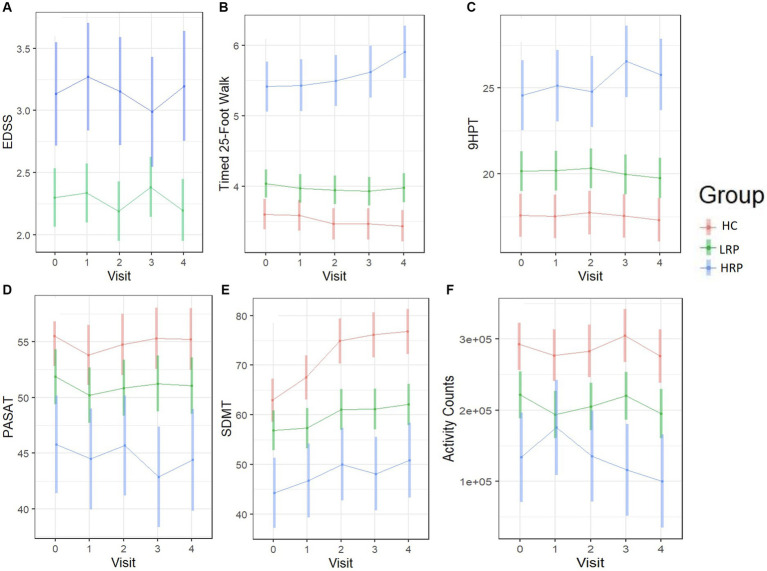
Longitudinal trajectories of clinical outcomes predicted by the fitted LME models. Color bars indicate 95% confidence intervals. **(A)** EDSS score (MS only). **(B)** T25FW (seconds). **(C)** 9HPT (seconds). **(D)** PASAT. **(E)** SDMT. **(F)** Daily activity counts (by ActiGraph). HC, healthy control; LRP, low risk progressor; HRP, high risk progressor. Missing data were 7, 14, 15 and 20% at four follow-ups.

**Figure 4:**
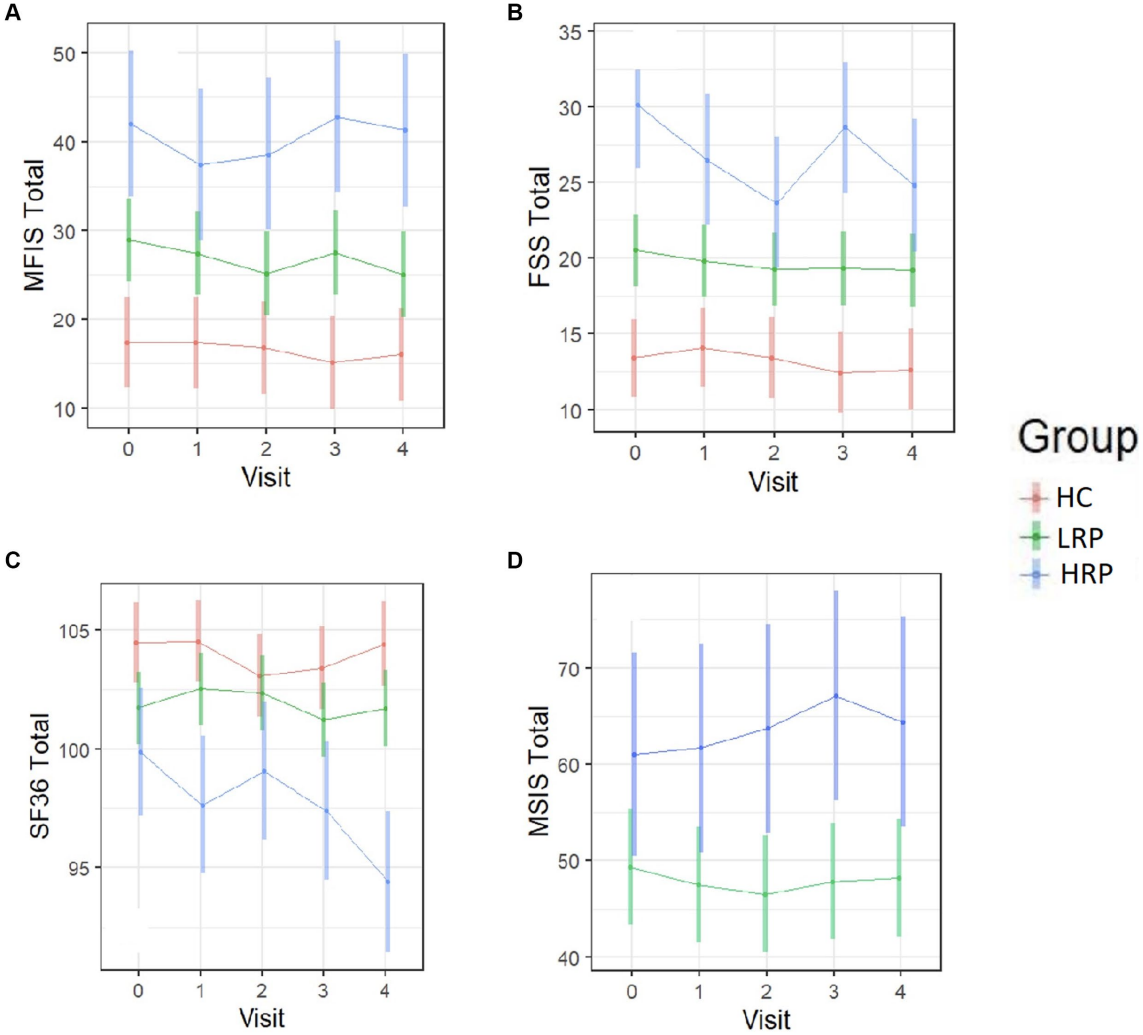
Longitudinal trajectories of PRO measures predicted by the fitted LME models. Color bars indicate 95% confidence intervals. **(A)** MFIS total score. **(B)** FSS total score. **(C)** SF-36 total score. **(D)** MSIS-29 total score (MS subjects only). HC, healthy control; LRP, low risk progressor; HRP, high risk progressor. Missing data were 6, 15, 15 and 19% at four follow-ups.

Using LME models, we predicted change from baseline across several clinical outcome measures among the three groups (HC, LRP, and HRP) (Supplementary Table S1). Although the HRPs had higher baseline EDSS, neither group had a change in EDSS over 2 years. Both the HC and LRP groups, but not HRP, had significant improvement in SDMT, demonstrating a learning effect that was relatively diminished in the HRP group. Only the HRPs had significant worsening on the T25FW and 9HPT. In contrast, LRPs demonstrated no progression on T25FW or 9HPT. Compared to HC, both LRPs and HRPs had a reduction in physical activity counts over time, with the greatest decrement seen in HRPs. Figure 3 illustrates by-group performance across the clinical outcomes. As expected, both MS subgroups underperformed relative to HCs, however, the HRPs demonstrated the poorest baseline and worsening performance longitudinally on all outcomes. The most notable changes were in the T25FW, 9HPT, and activity counts (Figures 3B,C,F). All groups had increased SDMT scores over time, however, the HRPs had attenuation of this learning effect relative to HCs and LRPs (Figure 3E). In addition, HRPs trended in worsening PASAT compared to LRPs (Figure 3D). Across PROs, HRPs similarly demonstrated the most significant progression (Supplementary Table S2). Only HRPs showed worsening from baseline on SF36 (*p* < 0.005), FSS (*p* < 0.005), and MSIS-29 (*p* = 0.06). Figure 4 illustrates predicted changes in PROs for the MS subgroups and HCs.

Overall, the multi-component CDP endpoint identified a total of 21 MS participants (34%) who demonstrated progression at 2-year timepoint. When compared against CDP, the proposed 6 MW^GST^ GMM approach had the best accuracy (71%), AUROC (0.67), and sensitivity (85%) among all clustering methods (Table 4). All clustering method had comparable PPV (around 75%) with the MSFC-based clustering showing exceedingly good PPV (81%). However, MSFC-based clustering had very low NPV (12%), whereas the 6 MW^GST^ approach had the best NPV (60%) among all methods. In other words, the 6 MW^GST^ approach was able to detect 60% LRPs correctly and 74% HRPs correctly given 34% prevalence of progression; whereas other tested approaches may not detect the LRPs as good as our 6 MW^GST^ approach.

**Table 4:** Comparison among different clustering methods with different predictors.

Clustering methods (Predictors)	AUROC (95% CI)	Accuracy (95% CI)	Positive predictive value	Negative predictive value	Sensitivity	Specificity
GMM (6MW^GST^)	0.67 (0.53–0.82)	0.71 (0.58, 0.82)	74%	60%	85%	43%
K-means (6MW^TD^ + 6 MW^min1^ + Δ6MW + Age + BMI)	0.61 (0.48, 0.73)	0.61 (0.47, 0.73)	75%	46%	58%	65%
K-means (T25FW + 9HPT + SDMT+PASAT + Age + BMI)	0.45 (0.33–0.57)	0.53 (0.40, 0.66)	81%	12%	58%	30%
K-means (Age + BMI + EDSS)	0.56 (0.44–0.68)	0.53 (0.40, 0.66)	73%	39%	46%	67%

## Discussion

Our work represents the first longitudinal study of the 6 MW gait speed trajectory (6MW^GST^) in MS and healthy control participants using the minute-to-minute walk distance during the 6 MW. Our findings indicate that the 6 MW^GST^ is a meaningful outcome in MS and confirms our prior cross-sectional validation study (9). Notably, on repeated 6 MWs, healthy controls increased their speed over the 2-years (Figure 2). MS participants demonstrated either no-change or a decrement in speed over the 2-years, LRP and HRP subgroups, respectively. Our 6 MW^GST^ approach to subgrouping has advantages over others found in the literature. Previously, others have focused on the difference between minute-6 and minute-1 of the 6 MW (often referred to as Δ6MW), for which specified cut-points are applied to identify MS subgroups (29, 30). In contrast, the 6 MW^GST^ integrates all six points of gait speeds and captures both within- and between-walk performance, allowing subgrouping without pre-defined and potentially erroneous cutpoints. We have recently demonstrated that the quadratic trajectories of the 6 MW (6MW^GST^), when modeled properly, provide more information than both total distance or the Δ6MW (9). Building on this work (9), we applied a GMM approach to integrating important 6 MW^GST^ information, including baseline gait speed and quadratic slopes of change.

Within our MS cohort, we present a novel method using the 6 MW^GST^ to stratify MS patients as having high or low risks of progression on a mix of clinical and patient-reported outcomes. Using the GMM method to cluster baseline 6 MW^GST^, we identified two MS subgroups with different risks of progression over the subsequent 2 years. Our two MS subgroups had distinctly different 6 MW^GST^ patterns which remained consistent over time, indicating that baseline 6 MW^GST^ features are unique and enduring within MS progressor subgroups. Only the HRP subgroup demonstrated progression across clinical (6 MW, T25FW, & 9HPT) and PRO measures (SF-36 & FSS) over 2 years. The HRPs also demonstrated attenuation in the SDMT learning effect seen on HC and LRPs. The learning effect in SDMT has been observed in others’ work (31, 32), and its attenuation in the HRP group in our study suggests a relative impairment of cognitive function. In addition, HRPs trended towards worsening on the PASAT, MSIS-29, and activity counts when compared to LRPs. In review of the literature, we note two studies that have looked at gait speed and its relationship with progression in MS with mixed results (33, 34). Muller et al. (34) utilizing wearable sensor technology during 6 MW reported no change in gait speed over a 12-month follow-up period in 50 MS and 20 healthy control study participants. In another 12-month study, Galea et al. (33) measured 6 MW gait speed using wearable sensor technology and found a significant decreased in gait speed over 12 months, but these changes were not reflected in the EDSS, which remained stable for most participants over the 12-month period. Similar to Galea et al. (33) we found that EDSS did not notably change over our 2-year study, despite other outcome measures capturing progression. The differences in findings across these two studies and our findings are likely multi-faceted and include notable differences between outcomes and study protocols. These include, method of 6 MW gait speed assessment, differences in the 6 MW protocol used, duration of follow-up, study eligibility criteria, and statistical analsyis method.

Collectively, findings support our hypothesis that the 6 MW^GST^-stratified HRP group experienced detectable and confirmed MS progression over 2 years. Signori et al. (35) applied latent class growth analysis on 10-year EDSS trajectories and identified three subgroups of MS disease progression (mild, moderate, severe). While they were able to group progression severity, they were unable to predict the risk to progress using only baseline data. Authors state, “*The lack of clearly distinct baseline characteristics among the three classes possibly reflects the inability to identify clear prognostic classes using baseline variables alone and highlights the presence of distinct but not predictable prognostic patterns using the set of baseline parameters used here*” (35). Our 6 MW^GST^ GMM approach presents a unique solution to identify risk of future disease progression. Importantly, although 6 MW^GST^ is a walking outcome measure, we confirmed progression in HRPs across several non-ambulatory outcomes. When comparing our stratification approach to others against 2-year CDP, our 6 MW^GST^ approach has better performance than using simple Δ6MW and total distance of 6 MW, as well as other commonly-used clinical outcome measures (e.g., EDSS or MSFC). Despite a low specificity (43%), the good sensitivity(85%), PPV(74%) and NPV (60%) of the 6 MW^GST^ approach indicates it has potential to be applied as a screening tool where sensitivity is preferred over specifity, such as MS clinical trials and research studies enrichment for participants with a high risk to progress is adventageous.

Currently, we lack a single “gold standard” of MS disease progression in the MS field. Although the CDP endpoint is routinely applied in MS research, the definition and implementation of CDP (e.g., included items) vary between studies. For example, CDP has been defined by EDSS alone, or integrated with MSFC components. In a recent, a pooled analysis of 23 MS trials CDP measured EDSS alone, identified only 7.2% of relapsing and 19.9–32.5% of progressive MS participants that progressed over time (36). Our MS participants had a mild-to-moderate disability at baseline (EDSS 1.0–4.0), which includes the EDSS range of lower sensitivity in capturing progression (37). Expectedly, in our study, the EDSS was insensitive to change over 2 years as a measure of disease progression. To overcome these limitations of the EDSS-only approach, we utilized the integrated EDSS and MFSC approach (EDSS-plus) (19, 38–40), including standard metrics for change in the EDSS, T25FW, and/or 9HPT to determine CDP. In our cohort, we identified 21 MS participants who meet this criterion, accounting for 34% of the MS group. Further, we found that the 6 MW^GST^ has good accuracy, sensitivity, and positive predictive value for CDP. In addition to CDP, across several validated MS outcome measures, HRPs demonstrated a clear, consistent pattern of progression relative to LRPs, validating our 6 MW^GST^ approach to stratifying MS participants into high and low risk groups for prorgression over 2-years.

Our study has some limitations. For example, our population was predominantly RRMS participants with mild-to-moderate disability. Future studies will be needed to complete external validation of the approach in both RRMS and larger progressive cohorts. Our longitudinal study followed participants for 2 years, which is a typical time-frame in MS research, but reperesents only a fraction of the disease duration. On average, MS patients can live with the disease for >25 years, and how these MS participants progression over a longer time horizon is not known. Nevertheless, the 2-year progression in PROs and MSFCs demonstrated in the identified HRP group provides a new approach which has important and relevant potential for application in MS research.

Halting MS disease progression represents a critical and unmet therapeutic need. Out of eight Phase III Trials in Progressive MS (41), only one study met its primary clinical endpoint and resulted in FDA drug approval (42). These recurring and disappointing results of progressive MS trials may, in part, be due to low on-study progression rates. Reliable methods are needed to enrich clinical trials with MS participants who are likely to progress within the timeframe of the study. Researchers have continued to work to identify improved outcome measures that may offer increased sensitivity in measuring disease progression (40, 43), while others have focused on integrated measures to predict future MS progression (44, 45). However, we currently lack an accessible method to prognosticate within-study progression. Leveraging the GMM approach, we have demonstrated that baseline 6 MW^GST^ can be used to identify MS subgroups with a high risk for disease progression over a 2-year horizon. Our work highlights the value of the 6 MW^GST^ as an additional MS outcome measure for progression prognosis. While predicting progression trajectory remains difficult, our subgrouping at baseline method without relying on longitudinal data is promising for predicting progression status and is an important first step towards improved prognosis. The 6 MW^GST^ represents a promising and sensitive tool for predicting the risk of MS disease progression with potential applications in both clinical care and clinical trials. Future research is needed to validate our findings in other MS cohorts, including those with a primarily progressive phenotypes.

## Data availability statement

The raw data supporting the conclusions of this article will be made available by the authors, without undue reservation.

## Ethics statement

The studies involving humans were approved by University of Virginia Institutional Review Board for Health Sciences Research. The studies were conducted in accordance with the local legislation and institutional requirements. The participants provided their written informed consent to participate in this study.

## Author contributions

MG: Writing – original draft, Writing – review & editing, Conceptualization, Data curation, Funding acquisition, Investigation, Methodology, Project administration. SC: Writing – original draft, Writing – review & editing, Formal analysis, Methodology. RM: Writing – review & editing, Conceptualization, Methodology, Resources. RP: Writing – review & editing, Data curation. UO: Writing – review & editing. JB: Writing – review & editing, Data curation.
