# Supplementary material for: Progression risk stratification with six-minute walk gait speed trajectory in multiple sclerosis
Source: Front Neurol. 2023 Oct 4;14:1259413. doi: 10.3389/fneur.2023.1259413 (PMC10582752; doi:10.3389/fneur.2023.1259413)
Supplement: Supplementary file 1 [file Table_1.docx]

**Supplemental Table S1**. Predicted changes from baseline in the three groups. Only MS subgroups analyzed for EDSS (MS specific outcome measure).

*Change from baseline to first follow-up is labeled as 0-1, and so on. ES indicate the values of change -- positive values indicate an increase and negative values indicate a decrease from baseline. Post-hoc multiple comparisons were adjusted by Benjamini-Hochberg correction. HC = Healthy Controls; LRP = Low Risk Progressors; HRP = High Risk Progressors.

|  |  | EDSS | | | PASAT | | | SDMT | | | T25FW | | | 9HPT | | | Activity Counts | | |
| --- | --- | --- | --- | --- | --- | --- | --- | --- | --- | --- | --- | --- | --- | --- | --- | --- | --- | --- | --- |
|  | Change from Visit 0 | ES | SE | p | ES | SE | p | ES | SE | p | ES | SE | p | ES | SE | p | ES | SE | p |
| HC | 1-0 |  |  |  | -1.71 | 0.80 | 0.06 | 4.57 | 1.57 | **0.01** | -0.01 | 0.07 | 0.86 | -0.07 | 0.28 | 0.81 | -15355 | 11975 | 0.24 |
|  | 2-0 |  |  |  | -0.75 | 0.84 | 0.43 | 11.94 | 1.65 | **<.0001** | -0.14 | 0.07 | 0.08 | 0.15 | 0.29 | 0.64 | -9476 | 12655 | 0.50 |
|  | 3-0 |  |  |  | -0.21 | 0.84 | 0.83 | 13.17 | 1.65 | **<.0001** | -0.14 | 0.07 | 0.08 | -0.04 | 0.29 | 0.90 | 12364 | 12668 | 0.38 |
|  | 4-0 |  |  |  | -0.27 | 0.85 | 0.80 | 13.83 | 1.67 | **<.0001** | -0.17 | 0.08 | **0.04** | -0.27 | 0.29 | 0.41 | -16515 | 13101 | 0.25 |
| LRP | 1-0 | 0.04 | 0.10 | 0.79 | -1.64 | 0.73 | **0.05** | 0.42 | 1.44 | 0.79 | -0.07 | 0.06 | 0.34 | 0.03 | 0.25 | 0.92 | -27824 | 10693 | **0.02** |
|  | 2-0 | -0.11 | 0.11 | 0.42 | -0.99 | 0.75 | 0.24 | 4.20 | 1.47 | **0.01** | -0.09 | 0.07 | 0.20 | 0.16 | 0.26 | 0.57 | -16535 | 10957 | 0.17 |
|  | 3-0 | 0.09 | 0.11 | 0.54 | -0.60 | 0.75 | 0.47 | 4.29 | 1.48 | **0.01** | -0.11 | 0.07 | 0.13 | -0.19 | 0.26 | 0.51 | -1494 | 11148 | 0.92 |
|  | 4-0 | -0.10 | 0.11 | 0.49 | -0.79 | 0.77 | 0.36 | 5.21 | 1.51 | **0.001** | -0.06 | 0.07 | 0.42 | -0.41 | 0.27 | 0.16 | -26479 | 11643 | **0.04** |
| HRP | 1-0 | 0.14 | 0.19 | 0.57 | -1.31 | 1.40 | 0.41 | 2.51 | 2.74 | 0.41 | 0.02 | 0.12 | 0.90 | 0.57 | 0.48 | 0.29 | 41944 | 24457 | 0.12 |
|  | 2-0 | 0.02 | 0.19 | 0.93 | -0.12 | 1.36 | 0.95 | 5.78 | 2.67 | **0.04** | 0.08 | 0.12 | 0.55 | 0.22 | 0.47 | 0.67 | 1827 | 22147 | 0.93 |
|  | 3-0 | -0.14 | 0.20 | 0.57 | -2.94 | 1.40 | 0.06 | 3.87 | 2.74 | 0.19 | 0.21 | 0.12 | 0.11 | 1.99 | 0.48 | **0.0001** | -17533 | 22939 | 0.49 |
|  | 4-0 | 0.06 | 0.20 | 0.80 | -1.40 | 1.44 | 0.39 | 6.59 | 2.82 | **0.03** | 0.49 | 0.13 | **0.0002** | 1.22 | 0.50 | **0.02** | -33585 | 23442 | 0.19 |

**Supplemental Table S2**. Predicted changes in PRO measures from baseline in the three groups.

|  |  | MFIS | | | FSS | | | SF36 | | | MSIS | | | | |
| --- | --- | --- | --- | --- | --- | --- | --- | --- | --- | --- | --- | --- | --- | --- | --- |
|  | Change  from Visit 0 | ES | SE | p | ES | SE | p | ES | SE | p | ES | SE | | | p |
| LRP | 1-0 | -1.49 | 1.46 | 0.36 | -0.69 | 0.724 | 0.39 | 0.80 | 0.72 | 0.33 | -1.88 | | 1.38 | 0.27 | |
|  | 2-0 | -3.78 | 1.5 | **0.02** | -1.25 | 0.742 | 0.12 | 0.63 | 0.74 | 0.45 | -2.86 | | 1.41 | 0.08 | |
|  | 3-0 | -1.43 | 1.5 | 0.39 | -1.18 | 0.743 | 0.14 | -0.49 | 0.74 | 0.57 | -1.57 | | 1.42 | 0.37 | |
|  | 4-0 | -3.89 | 1.54 | **0.02** | -1.31 | 0.761 | 0.11 | -0.01 | 0.76 | 0.99 | -1.17 | | 1.45 | 0.50 | |
| HRP | 1-0 | -4.62 | 2.8 | 0.13 | -3.63 | 1.385 | **0.01** | -2.22 | 1.37 | 0.17 | 0.71 | | 2.65 | 0.82 | |
|  | 2-0 | -3.45 | 2.8 | 0.26 | -6.46 | 1.385 | **<.0005** | -0.80 | 1.37 | 0.61 | 2.71 | | 2.65 | 0.41 | |
|  | 3-0 | 0.77 | 2.79 | 0.82 | -1.48 | 1.381 | 0.33 | -2.47 | 1.37 | 0.13 | 6.09 | | 2.64 | 0.06 | |
|  | 4-0 | -0.75 | 2.88 | 0.82 | -5.32 | 1.426 | **<0.005** | -5.47 | 1.41 | **<0.005** | 3.40 | | 2.73 | 0.32 | |

*Change from baseline to first follow-up is labeled as 0-1, and so on. Positive values indicate a increase and negative values indicate an decrease from baseline. Post-hoc multiple comparisons were adjusted by Benjamini-Hochberg correction. LRP = Low Risk Progressors; HRP = High Risk Progressors.
